# CUDAMPF: a multi-tiered parallel framework for accelerating protein sequence search in HMMER on CUDA-enabled GPU

**DOI:** 10.1186/s12859-016-0946-4

**Published:** 2016-02-27

**Authors:** Hanyu Jiang, Narayan Ganesan

**Affiliations:** Department of Elec. and Comp. Engg, Stevens Institute of Technology, Hoboken, NJ 07030 USA

**Keywords:** SIMT, SIMD, CUDA, Hidden Markov model, Parallelism, Single segment Viterbi, Multiple segment Viterbi, Viterbi

## Abstract

**Background:**

HMMER software suite is widely used for analysis of homologous protein and nucleotide sequences with high sensitivity. The latest version of *hmmsearch* in HMMER 3.x, utilizes heuristic-pipeline which consists of MSV/SSV (Multiple/Single ungapped Segment Viterbi) stage, P7Viterbi stage and the Forward scoring stage to accelerate homology detection. Since the latest version is highly optimized for performance on modern multi-core CPUs with SSE capabilities, only a few acceleration attempts report speedup. However, the most compute intensive tasks within the pipeline (viz., MSV/SSV and P7Viterbi stages) still stand to benefit from the computational capabilities of massively parallel processors.

**Results:**

A Multi-Tiered Parallel Framework (CUDAMPF) implemented on CUDA-enabled GPUs presented here, offers a finer-grained parallelism for MSV/SSV and Viterbi algorithms. We couple SIMT (Single Instruction Multiple Threads) mechanism with SIMD (Single Instructions Multiple Data) video instructions with warp-synchronism to achieve high-throughput processing and eliminate thread idling. We also propose a hardware-aware optimal allocation scheme of scarce resources like on-chip memory and caches in order to boost performance and scalability of CUDAMPF. In addition, runtime compilation via NVRTC available with CUDA 7.0 is incorporated into the presented framework that not only helps unroll innermost loop to yield upto 2 to 3-fold speedup than static compilation but also enables dynamic loading and switching of kernels depending on the query model size, in order to achieve optimal performance.

**Conclusions:**

CUDAMPF is designed as a hardware-aware parallel framework for accelerating computational hotspots within the *hmmsearch* pipeline as well as other sequence alignment applications. It achieves significant speedup by exploiting hierarchical parallelism on single GPU and takes full advantage of limited resources based on their own performance features. In addition to exceeding performance of other acceleration attempts, comprehensive evaluations against high-end CPUs (Intel i5, i7 and Xeon) shows that CUDAMPF yields upto 440 GCUPS for SSV, 277 GCUPS for MSV and 14.3 GCUPS for P7Viterbi all with 100 % accuracy, which translates to a maximum speedup of 37.5, 23.1 and 11.6-fold for MSV, SSV and P7Viterbi respectively. The source code is available at https://github.com/Super-Hippo/CUDAMPF.

## Background

Protein motif detection is key to identifying conserved protein domains within family of proteins as well as deducing its structure and function within the genome. The HMMER [1, 2] suite of programs is widely used for protein motif finding, building the profiled Hidden Markov Model (HMM), scanning an entire database of HMMs for all motifs etc. The current version, HMMER ver 3.x, is a significant improvement over its predecessors due to the scoring system used to compute the statistical significance of alignment scores. Among the suite of tools in HMMER, hmmsearch is used to detect a query motif among a target database of sequences. The wide applicability of motif finding, the rapid growth of the set of protein families as well as the set of known sequences has made it target of many acceleration attempts. Although the list of acceleration attempts for HMMER 2.x [3] is not exhaustive, some representative contributions include [4–11]. While HMMER 2.x used Viterbi algorithm (for optimal alignment) to compute the scores, HMMER 3.x follows a scoring system that computes the total log-likelihood ratios summed over all possible alignments, via the Forward-Backward algorithm [2]. Optimal alignment scores are useful in studying similarity between individual sequences (as in BLAST [12] or Smith-Waterman [13] algorithms for local alignment), the Forward scores are more meaningful in alignment of target protein sequences against a probabilistic model such as the HMM.

Although the Forward-Backward algorithm for probabilistic inference has the same computational complexity as the Viterbi algorithm, computing the Forward scores requires much higher computational throughput (FLOPS) than the Viterbi algorithm [2]. This is due to the sensitivity of the sequential dependency imposed by the D-D transitions in the profiled HMM. The D-D transitions in the Forward algorithm are always essential to computing the overall scores, where as the D-D transitions in Viterbi algorithm has an effect, only if it scores higher than the other transitions. This enables various methods to quickly assert the impact of D-D transitions in Viterbi scores and increase its overall throughput, and are not applicable to accelerating Forward score computation.

It is shown that [3] distribution of high-scores of optimal alignment (via Viterbi algorithm) is Gumbel distributed with parameter *λ*= log2 and that of Forward scores (total log-likelihood ratio sums) is exponentially distributed with the same *λ*= log2. Hence, the high-scoring tails of Viterbi and Forward scores agree with each other, which enables designing an efficient task pipeline that can filter out sequences based on Viterbi scores that are not expected to score high via the Forward algorithm. Although, this pipeline removes the load off the Forward score computing stage, the Viterbi based pre-filtering is still as expensive as the scoring system employed in HMMER 2.x. In order to mitigate the computational workload on the P7Viterbi stage a heuristic Multiple-Segment-Viterbi (MSV) is introduced that is analogous to word hit and ungapped extension stages implemented in BLAST. The MSV stage employs a much simpler Hidden Markov Model for scoring that eliminates sequential dependencies between the Dynamic Programming (DP) matrix cells which was “Vectorized” on a parallel machine. Through choice of sensitivity parameters of MSV scores in HMMER 3.x, an 8-bit saturating scoring system was used whose computation was vectorized on a 128-bit SSE register as 16 parallel operations on 8-bit data, thus achieving a 16-fold speedup on a commodity processor core. Furthermore, the latest version of HMMER, ver 3.1, includes the SSV(Single ungapped Segment Viterbi) sub-stage, another heuristic to accelerate the MSV stage by ignoring “*J*” state transitions that is designed to chain multiple matches together [14]. Although this may result in false negatives on the score of sequences, it provides speedup over MSV significantly.

### Previous work

Due to extensive computational and scoring optimization procedures implemented in HMMER 3.x [2], it is extremely unlikely to improve the performance further either on CPU or GPU based platforms with generic optimization techniques alone. For the previous version, HMMER 2.x, which is based on Viterbi algorithm, several strategies were proposed to accelerate the underlying Viterbi score calculation. hmmsearch in HMMER 2.x was initially parallelized for clusters via MPI in [4] where the state loop was vectorized to process 24 HMM states in SIMD fashion or 8 state triplets at once. The initial work utilizing Graphics Processing Units (GPUs) to accelerate hmmsearch in HMMER 2.x is Claw-HMMER [5], and GPU-HMMER [6] achieves limited speedup over it. Partial prefix sums were used [7] to break the chain of dependencies in computation of Viterbi scores. This helped extract a hybrid task and data-level parallelism in order to solve the load imbalance problem that arises due to variations in sequence lengths. In addition to multiprocessor systems, a number of attempts to accelerate implementation of the HMMER recurrence have been carried out for FPGAs [8–10]. An extensive review of various acceleration attempts was compiled in [11].

However, unlike the previous version, which has been target of numerous acceleration attempts, there exist only a handful of existing work aimed to improve the performance of key segments of HMMER 3.x pipeline. The main reason being HMMER 3.x is already highly optimized and is about 100- to 1000- fold faster than HMMER 2.x [1], implemented on the commodity processors with SSE support and multi-core parallel. This renders any acceleration attempt for previous versions of HMMER obsolete. Hence alternative architectures such as FPGA [15] have been explored as an accelerator hardware for MSV and P7Viterbi segments in HMMER 3.x. The Viterbi algorithm was rewritten for parallelization via prefix sums approach on the FPGA and is able to achieve comparable performance for P7Viterbi implemented on dual-core processors. However the hardware limitations on the FPGA makes this implementation suitable for smaller models (upto 512) and tiling larger models into several dataflow partitions.

In [16], a speculative GPU based method was implemented to reduce the global memory access within the kernel of MSVFilter. This approach aims to reduce the execution time of original reduction loop empirically. Lin’s work [17, 18] also focused on MSV stage by following the parallel strategy of GPU-HMMER but introducing SIMD instructions. Different sequences were assigned to individual threads in both methods. Partial optimization was proposed in [19], which parallelizes the P7Viterbi part without considering the *D*-*D* path dependency. Although this approach claims a 14x speedup than original functions, it sacrifices the sensitivity of probabilistic inference. Another attempt of accelerating P7Viterbi [20] was implemented on Intel and AMD CPUs with proposed cache-oblivious strategy that offsets cache miss penalties of original work. Moreover, the newest stage of HMMER 3.1, SSVFilter, was accelerated [21] through a set of optimizations that mainly include model tiling, loop unrolling, coalesced and vectorized memory access.

Other work related to pairwise and multiple sequence alignment based on the Smith-Waterman and Needleman-Wunsch algorithms have been accelerated on CUDA-based GPUs. CUDA-LINSi [22], a Multiple Sequence Alignment (MSA) algorithm, accelerated CPU-based LINSi of MAFFT [23] software package by optimizing global and shared memory access as well as employing data compression techniques. Pairwise and group-to-group alignments are calculated by individual threads in this work. Another CUDA-based MSA acceleration, CUDA ClustalW [24], assigned one pairwise alignment to a thread block by extracting parallelism along the major/minor diagonal direction. [25] proposed a comprehensive acceleration solution on the GPU for all-to-all pairwise global, semi-global and local sequence alignments, using tile-based dynamic programming framework that significantly reduces the number of write/fetch through device memory. However, in contrast to sequence alignment algorithms, protein-motif finding imposes non-local and complex dependencies between the dynamic programming (DP) cells which necessitates alternative techniques for parallelization.

### CUDA-enabled GPU architecture

As a parallel computing engine, CUDA-enabled GPUs are built around a scalable array of multi-threaded streaming multiprocessors (SM) for large-scale data and task parallelism, which are capable of executing thousands of threads based on SIMT mechanism. Following Tesla [26], Fermi [27] and Kepler [28] to latest Maxwell [29], each generation has more hardware resources and newer intrinsic functionalities than the previous. Our proposed algorithms and methods in this paper which utilizes the latest intrinsics, are designed for the Tesla K40 of Kepler architecture with compute capability 3.5 or higher. The Kepler architecture also features more powerful streaming multiprocessor (SMX) which consists of 192 single-precision CUDA cores, 64 double-precision units, 32 special function units and load/store units [28]. In highlight, the architecture offers another 48 KB on-chip Read-Only Data cache with an independent datapath from the existing L1 and shared memory datapath, and the maximum amount of available registers for each thread is increased to 255 for GK110 instead of prior 63 per thread.

### HMMER pipeline: MSV/SSV and P7Viterbi

**Fig. 1:**
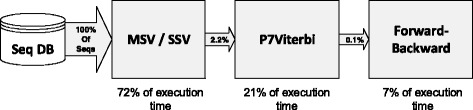
Profiled HMM models. (**a**) MSV model; (**b**) SSV model; (**c**) P7Viterbi model

**Fig. 2:**
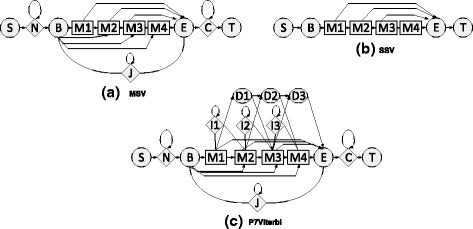
Heuristic pipeline of HMMER 3.x. A sample benchmark with query model of 400 length and the Env-nr database consisting of 6.5 million protein sequences

The task pipeline of HMMER 3.x is optimized for computational efficiency that employs heuristics to eliminate vast majority of low scoring sequences by introducing MSV- and SSVFilter. As sequences filters, MSV detects contiguous match alignments while SSV captures single match aligmnet, which are analogous to the ungapped high scoring pairs implemented in BLAST. Although BLAST uses a two-stage filter to detect and extend the ungapped alignments, the uniform entry/exit probability in the MSV/SSV model allows for partial matches upto the size of the query motif. The profile MSV/SSV models are shown in Fig. 1(a,b), and the full Plan-7 Viterbi model (P7Viterbi) is shown in Fig. 1(c). Without inter-row dependency caused by “*J*” state, the potential missing of higher score will be checked in SSV stage [14] followed by a regular MSV processing. As shown in Fig. 2, the result of pipeline benchmark indicates that 2.2 % of the sequences cross the MSV/SSV threshold to be passed on to the P7Viterbi stage. Only 0.1 % of all the sequences are passed on to the Forward-scoring stage. The corresponding execution time is close to 72 % for MSV/SSV, 21 % for P7Viterbi and 7 % for Forward-Backward stage.

## Methods

### GPU acceleration

Since the majority of the execution time is spent in the MSV/SSV filtering stage, it is a prime candidate for acceleration. As vast majority of input sequences are also eliminated in this stage, any improvement in the performance will greatly impact the efficiency of the pipeline. Both MSV and SSV model exhibits regular and well-behaved dependencies that can be easily parallelized compared to P7Viterbi. However, in order to exceed the performance of the highly optimized MSV/SSV filter of latest HMMER especially on multi-core CPUs, it is imperative to go beyond generic parallelization techniques and exploit architecture-specific intrinsics.

The model simplifications in the MSV compared to the full core model used in P7Viterbi stage eliminates the “*Delete*” states that induce sequential dependencies between the cells of the dynamic programming matrix within each row. The “*Insert*” states that induce dependencies to the previous rows are also eliminated, leaving only the “*Match*” states that induce a diagonal dependency to cells in the previous row. SSV model, additionally removes the “*J*” state which eliminates a portion of heavy workload within the computationally intensive innermost loop. However, most of existing work with coarse-grained parallelization ignore the overhead caused by synchronization within the MSV/SSV and P7Viterbi kernels on GPU, which forces active threads to enter idle state and wait for other threads to complete. The problem is further amplified by the fact that the total number of alignment, each with multiple synchronizations, is equal to the total number of collective residues contained within all sequences (typically billions of residues), which can severely limit the performance. Further optimization attempt must avoid unnecessary synchronization or totally eliminate them if possible.

#### Warp-synchronous execution

With the current SIMT mechanism of CUDA-enabled GPUs, we exploit the fact that every 32 threads within a thread-warp are always executed synchronously by the current CUDA programming model. Thus, we make each warp processes a sequence residue by covering a single row of the DP matrix moving on to the successive row (next residue) of the sequence until the entire sequence is scored. Hence by having a single warp update all the cells within each row, the need for synchronizations can be eliminated. Moreover, in order to avoid data dependency between warps, each thread-warp processes a different sequence and continues to process the next sequence in the database independent of other warps within the SMX or the device. This again eliminates need for any block-level coordination or stalling due to synchronization, and helps keep active threads always busy and maximize kernel throughput. This achieves true independence between warps and completely eliminates the demand of synchronization throughout the course of entire execution.

#### CUDA-SIMD based parallellization

In addition to CUDA C/C++, CUDA-enabled GPUs also support a low-level programming model via parallel thread execution (PTX) virtual machine with instruction set architecture (ISA) [30], for efficient data-parallel computing. Since PTX ISA version 3.0, a set of SIMD (Single Instruction Multiple Data) video instructions has been introduced for intra-word operations such as quads of 8-bit values and pairs of 16-bit values. Table 1 lists all SIMD video instructions that are hardware accelerated for Kepler architecture and only available on devices of compute capability 3.0 or higher [31]. In this work, we increase the parallel throughput by embedding SIMD intrinsics within warp-based, self-synchronous SIMT mechanism of GPUs. Similar to the SSE (Streaming SIMD Extensions) instruction set on CPU, which supports 128-bit registers with 16-lane parallelism, the SIMD video instructions on the GPU enable 4-lane data parallelism per thread. This increases the available parallelism within a single-warp from 32 to 128, all of which are executed without any synchronization overhead. By assigning each sequence to individual warps, both the parallel throughput as well as hardware resource utilization are greatly enhanced. This finer-grained parallelism helps obtain augmented speed-up on CUDA-enabled GPUs and introduces a new tier of parallelization.

**Table 1:** Integer half-word/quad-byte SIMD video instructions

Intrinsic PTX assembly	Semantics	Operands and
		Optional operations
vadd2, vsub2, vadd4, vsub4	Addition/Substraction	.u32.s32.sat.add
vmax2, vmin2, vmax4, vmin4	Maximum/Minimum	.u32.s32.sat.add
vset2, vset4	Comparison	.u32.s32.cmp.add
vavrg2, vavrg4	Average	.u32.s32.sat.add
vabsdiff2, vabsdiff4	Absolute value of difference	.u32.s32.sat.add

Respectively, u32 and s32 represent unsigned and signed values of 32-bit; sat is used to clamp the range of operand based on its bit-width; add is for accumulation; cmp consists of 6 comparison operators: eq, ne, lt, le, gt, ge

#### GPU runtime compilation

In compiled programs the parameters defined at compile-time via macro constants make various compiler optimizations possible. However, most of values of those parameters are only known at runtime, thus disabling the compiler to optimize kernel as much as possible at offline compile-time. Runtime compilation enables programmer to take advantage of improved performance due to predefined macro parameters but in run-time. In addition, it also enables application driven construction of the kernel dynamically at runtime. Based on the knowledge of the problem data, it is possible to dynamically construct various parts of the kernel from a repertoire of subparts, optimized for the current problem. In this work the advantage offered by NVIDIA Runtime Compilation, NVRTC, was leveraged in order to define the HMM and database parameters at runtime so as to enable compiler optimizations such as loop unrolling, as well as switchable kernels, to increase data locality and pursue better performance.

### Four-tiered parallelism

**Fig. 3:**
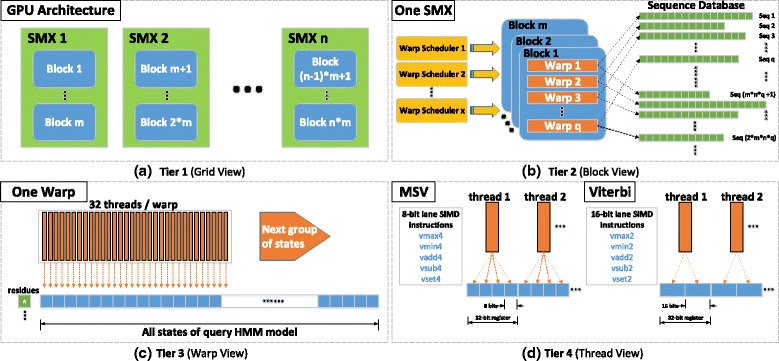
CUDAMPF: Multi-tiered Parallel Framework on CUDA-enabled GPU. (**a**) A single GPU consists of *n* SMXs with *m* concurrently mounted blocks on each; (**b**) within each block, *q* resident warps are scheduled by *x* warp scheduler for processing assigned sequences; (**c**) a warp of threads score alignment of all residues and model states in parallel (warp size is fixed to 32 currently); (**d**) based on 32-bit register and score ranges of different algorithms, each thread processes multiple model states in a single step. The virtual boundary, *block*, is only regarded as the container of warps rather than a separate tier

On a single GPU, Multi-tiered Parallel Framework (CUDAMPF) is organized into four tiers of parallelism, comprising of SIMT and SIMD execution. The top three tiers, derive from our previous work [32], are based on SIMT mechanism and the last tier is built on SIMD intrinsics. Figure 3 gives a overview of the framework: the first tier of parallelism is built on the multiple SMXs that work concurrently; the second tier is composed of multiple resident warps within each SMX, that process different sequences and are independently driven by multiple warp schedulers; the third tier is due to the warp synchronism, where all threads of the same warp compute alignment scores of the same sequence concurrently; the fourth tier is built on SIMD intrinsics, where every thread operates on quads of 8-bit values for MSV and pairs of 16-bit Viterbi scores (cells of the dynamic programming matrix) respectively. The multiple tiers are oblivious to the CUDA version and are only related to hardware resources and device properties such as the number of SMXs, warp size and bit-width of registers that are simply queried at runtime and provided as built-in constants. This kind of hardware-aware optimization makes the parallelization scalable and portable thus fully utilizing the computational capability of CUDA-enabled GPUs.

There is no demand of explicit thread-synchronization to keep the threads consistent within the same thread-block. Across all SMX units, warps process their own sequences proceeding to the next target upon completion, independently. The index of next sequence for each warp is calculated as:

(1) $$ {I}_{next}={O}_{warp}+{C}_{warp}\times{N}_{warp}\times{N}_{smx}  $$

where *O*_*warp*_ is the ordinal ID of a warp across *N*_*smx*_ SMXs, *C*_*warp*_ is a counter that records the number of sequences processed by this warp, *N*_*warp*_ is the total number of resident warps per SMX on the launched kernel. Warps always keep selecting the next sequence as long as the index *I*_*next*_<*TOTAL*, the total amount of sequences within database. Without any request of synchronization, CUDAMPF avoids thread idling caused by unbalanced length of sequences as well as correctness check across boundary due to concurrency and racing hazard amongst threads, which improves speed and throughput.

### Implementation details

#### Striped layout vs. sequential layout

**Fig. 4:**
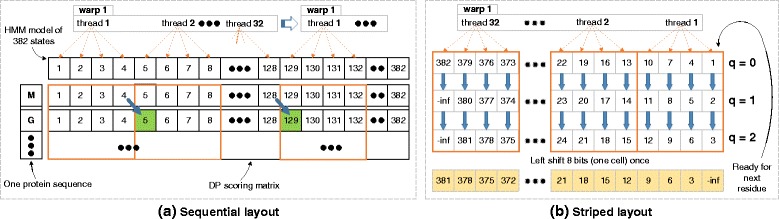
Comparison of alignment with sequential and striped layout. An example of MSV score alignment with HMM model of *L*_*hmm*_ = 382. (**a**) 32 threads of *w* *a* *r* *p*1 concurrently calculate 128 8-bit scores at each iteration, and every 4 scores are stored together as one 32-bit datatype. Orange-colored boundaries indicate private regions of different threads, where threads only take charge of cells within their own regions. (**b**) a warp of threads process model states with striped interval *Q*=3 that is also the number of iteration required for the current row. Yellow-colored cells represent the result after one 8-bit shifting from *q*=*Q*−1=2, which will be used to calculate *q*=0 for next residue

Although sequential layout of dynamic programming cells is straightforward, it is not suitable for SIMD operations in the presence of diagonal dependencies between DP cells such as in MSV/SSV and Viterbi algorithm: Each thread is dependent on the value computed by an adjacent thread (as shown in Fig. 4(a)), in the previous iteration. This is because using per thread memory such as registers or local memory will require extra instructions like shifting and bitwise operations to exchange private data (i.e, score of state 5 is private for thread 2) between threads. Moreover, calculating score of the first cell (i.e, score of state 129 needs state 128 in the previous iteration) after each iteration imposes additional sequential overhead. These overmuch instructions and thread idling result in weak parallelism that will be further ampilified within the innermost loop.

In order to avoid pitfalls of sequential layout on performance, a striped layout similar to the SSE implementation in HMMER is adopted, but across all threads within the entire warp. This proposed layout of scoring alignment for GPU kernel is shown in Fig. 4(b), which does not impose any dependencies between cells. Each thread calculates four or two scores (cells) concurrently with striped intervals *Q* that is defined as:

(2) $$ Q = \text{max}\left(\frac{({L}_{hmm}-1)}{{\alpha}_{algor.}\times{S}_{warp}}+1, 2\right)  $$

where *L*_*hmm*_ is the length of query model, *S*_*warp*_ is the size of warp and *α*_*algor*._ indicates lanes of parallelism for specific algorithm (i.e. *α*_*msv*/*ssv*_=4, *α*_*viterbi*_=2). By coupling SIMD instructions and SIMT mechanism, each thread handles multiple striped sub-words concurrently (i.e, states 1, 4, 7 and 10 are stored together as a 32-bit datatype and are always calculated by thread 1, as shown in Fig. 4(b)). In contrast with *hmmsearch* ran on the SSE-supported CPU which achieves only 16-fold and 8-fold [2] parallelism, our proposed layout achieves 128-fold parallelism for MSV/SSV and 64-fold parallelism for Viterbi algorithm on a GPU: each warp calculates 128 or 64 scores in parallel and iterates *Q* times to finish each row of the DP matrix. After *Q* iterations, only one parallel reordering across a warp of threads is needed to satisfy the diagonal dependency for the next DP row, which guarantees every thread always process same states of the query model. Private registers and local memory of each thread are able to be frequently reused for scoring alignment in the case.

In addition, the *transition* and *emission* parameter matrices are also pre-formatted to be the same striped layout (either as four 8-bit scores or two 16-bit scores packed in one) and are stored contiguously for the indexing by a warp of threads during iterations of innermost loop. As the number of cells per DP row is fixed (i.e, 128 or 64 cells, totally 128B width), any query model with the size that is not an integral multiple of 128 or 64 will be padded with the initial value, −*inf* (0, –128 or –32768). This results in coalesced access to off-chip memory with only one transaction per memory request.

#### MSV/SSV and Viterbi algorithm with SIMD

Algorithm 1 outlines the main structure of MSV kernel with three loops. *Loop* A (Tier 2) iterates over the different target sequences for each warp and *Loop* B (Tier 3) iterates over all residues of current sequence. In order to decrease the latency of sequence read, the sequence data is pre-fetched from global memory into the shared memory buffer of size *S*_*warp*_×4 per warp, where each thread fetches 4 residues packed into a 32-bit word as shown in line 4. As a result, every iteration of *Loop* B process up to 128 residues with only one coalesced global memory transaction. The innermost loop, *Loop* C (Tier 4), is the last tier with embedded SIMD instructions within the SIMT mechanism. The underscored SIMD instructions, *vmaxu*4,*vaddus*4 and *vsubus*4, represent per-byte unsigned maximum, saturated addition and subtraction with values clamped to 0 and 255. SSV algorithm, as shown in Algorithm 2, is easily implemented under the proposed framework like MSV kernel. However, it removes all calculations related to *xJ* and *xB* which allows significant speedup inside *Loop* C.





Algorithm 3 shows the outline of the P7Viterbi segment, which follows the same general framework. However the presence of *Match(M)* and *Insert(I)* states introduces additional dependencies between successive iterations and the presence of *Delete(D)* states imposes sequential dependencies within the same iteration. The *D*-*D* dependencies imposed by the *Delete* state is resolved via the *Lazy*-*F* method introduced in [33], also implemented in *hmmsearch* and is shown from lines 26–42. The value of register *R*_*dcv*_ will be sent to *Loop* E after re-ordering to check for potentially higher scores across *Q* cells (the column of striped layout). By coupling SIMD and SIMT on GPU, compared to 8-fold parallelism of SSE based *hmmsearch*, a 64-fold parallelization per warp is achieved to accelerate parallel *D*-*D* checking of *Lazy*-*F* in a finer-grained way that largely eliminates sequential overhead.



#### Reordering and max-reduction for SIMD & SIMT

**Fig. 5:**
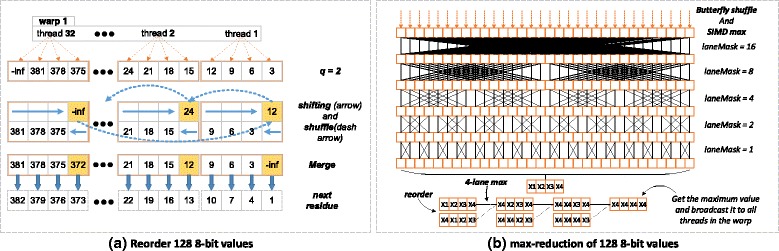
Illustrations of proposed reordering and maximum functions for CUDAMPF. Assuming *x*4>*x*3>*x*2>*x*1 after intra-warp reductions in (**b**)

**Fig. 6:**
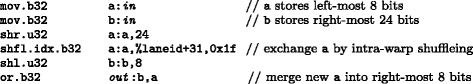
Pseudo PTX assemblies of inline reordering function. An example of reordering 128 unsigned values of 8-bit for MSV kernel whereas P7Viterbi kernel will process 64 signed values of 16-bit

In order to implement the striped layout with SIMD & SIMT, a parallel reordering of all 8-bit or 16-bit values amongst intra-warp threads is necessary at the last iteration step *q*=*Q*−1. As illustrated in Fig. 5(a), we proposed an inline function of PTX assembly, *reorder*_*uint*8 (line 6 in Algorithm 1), that extracts one sub-word value from private memory of each thread, exchanges it through intra-warp shuffling as a closed cycle (i.e. *thread*32 sends its private data to *thread*1) and then merges this exchanged value into private memory space again. This procedure is completely concurrent for each warp in which case all threads inside are active, and the details of this inline function are depicted in Fig. 6. Proposed SSV algorithm shares the same idea but needs shift in 0×80 instead of 0 as −*inf*.

**Fig. 7:**
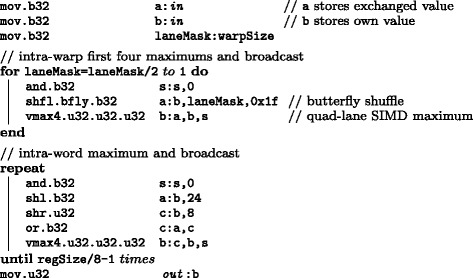
Pseudo PTX assemblies of inline maximum reduction function. An example of maximum reduction for MSV kernel. s is an auxiliary register used with vmax instruction

Another function of PTX assembly for maximum reduction, *maxred*_*uint*8 (line 15 in Algorithm 1), across all the threads within the warp is required to compute the terminal cost, *xE* of each row of the DP matrix. As shown in Fig. 5(b), butterfly shuffling and quad-lane SIMD maximizing make sure that large values always be broadcasted to all threads at each step. After five reductions due to *S*_*warp*_=32 fixed by current CUDA model, every thread then keeps four largest values (1st, 2nd, 3rd and 4th) that are packed with 32-bit datatype. And the last step is intra-word shifting with the SIMD maximum instructions to obtain and broadcast the maximum value (the largest one of 128 or 64 *xE*s). Figure 7 gives pseudo PTX assemblies for this maximum reduction.

As to Viterbi algorithm, functions with *int*16 suffix shown in Algorithm 3 indicates the width of sub-word is increased to 16-bit that reduces the number of reductions as well as shifting bits. These inline functions of PTX assemblies enable Tier 4 in CUDAMPF to be feasible with our proposed striped layout, and also eliminate the overhead of accessing shared memory compared to intra-register operation.

#### Hardware-aware resource allocation

Although the multi-tiered parallelization is designed to take advantage of the execution model on massively parallel processors, in order to fully leverage the power of the underlying hardware, it is necessary to maintain and optimize the device resources, like the on chip and off chip memory/cache system, with full awareness of their capabilities and performance. Any improper allocation strategy would not only impair performance but also limit the scalability with respect to the data size.

In the previous work [32] we evaluated the performance by using shared memory and global memory to store score matrices of model and alignment scores, where both of them met limited speedup with large query model that reduced the number of resident warps on each SMX. To solve the trouble of scalability, present work uses register and local memory to store all alignment scores like *MMX*, *IMX*, *DMX* in Algorithms 1 and 3. This benefits from Kepler architecture that supports available local memory space for each thread *N*_*local*_=512 KB at most [28], which enables *L*_*hmm*_ upto $\left (\frac {({N}_{\textit {local}}\times 1024)}{4 B\times 3}-1\right)\times {\alpha }_{\textit {viterbi}}\times {S}_{\textit {warp}} + 1$ theoretically. Although local memory is off-chip memory, it is naturally organized as a layout of consecutive 32-bit words accessed by consecutive threads [31], that is well consistent with our striped layout of scoring alignment since all threads within a warp access their score arrays *M*/*I*/*DMX*[ *i*] with same index. This enable our warp-based operations (at 3rd tier) always archieve coalesced access to local memory with 128-bytes memory transactions, which is the optimal accessing pattern.

As this strategy leaves most of the memory on-chip unused, 48 KB of it can be configured to serve as L1 cache, thus improving cache hit ratio and performance. Furthermore, the HMM model parameters such as the emission and transition scores, *E*_*msv*/*ssv*_, *E*_*vit*_ and *T*, can be stored in the global memory and cached by the 48 KB of Read-Only cache, as the parameter values are fixed throughout the course of the application. The use of on-chip shared-memory for storing the HMM parameters is not beneficial because (a) large models cannot fit within limited size of the memory and (b) the indeterminate access pattern to the parameter matrix stored in the shared memory as dictated by current residue will lead to bank conflicts and loss of performance. However, the case of register spill and cache hit ratio become impact factors of performance now. For the register-intensive kernel, more active threads will tighten the amount of available registers to each thread that leads to severe register spill. Escpecially for P7Viterbi kernel, much more local memory and registers are consumed in comparison to MSV/SSV kernel, that is not only caused by additional *D* and *I* scores but also due to instruction complexity of *Loop* C. An effective solution is properly reducing the quantity of threads that makes each of them obtains more assigned registers clamped by compiling option “-maxrregcount”. We empirically launched 32 resident warps per SMX for both MSV/SSV and P7Viterbi kernels, and it obtained good trade-off with high performance.

This work also examines the performance of using on-chip shared memory to store alignment scores. Given two built-in parameters *S*_*shared*_ and *S*_*thread*_ as maximum amount of shared memory and resident threads per SMX respectively, the relationship between optimal occupancy *P* and the usage of shared memory per SMX, *U*_*msv*/*ssv*/*vit*_, can be described as:

(3) $$ \begin{aligned} & {U}_{msv/ssv}=(Q+1)\times{S}_{warp}\times{4}\times\hat{N}_{warp} \\ & {U}_{vit}=(3\times{Q}+1)\times{S}_{warp}\times{4}\times\hat{N}_{warp} \\ & P=\frac{\hat{N}_{warp}\times{S}_{warp}}{{S}_{thread}}\times{100}\% \\ \end{aligned}  $$

with two constraints: $\hat {N}_{\textit {warp}}\times {S}_{\textit {warp}}\le {S}_{\textit {thread}}$ and *U*_*msv*/*ssv*/*vit*_≤*S*_*shared*_ where $\hat {N}_{\textit {warp}}$ is the maximum amount of resident warps can be launched. Increasing the length of model, *P* decreases rapidly due to $\hat {N}_{\textit {warp}}$.

#### Selective kernel compilation and loop optimizations via NVRTC

**Fig. 8:**
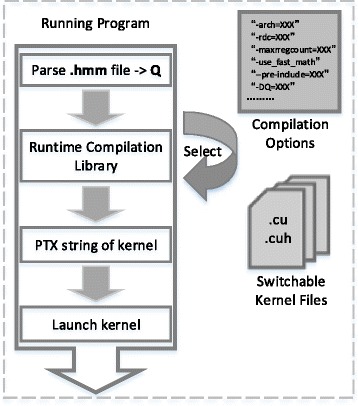
CUDAMPF program with NVRTC. After obtaining query model size and device properties, program dynamically makes decisions on unrolling innermost loop and selects the proper kernel file with compiler options

In CUDA 7.0, a runtime compilation library NVRTC is introduced to dynamically compile CUDA kernel source against offline static compilation [34]. This is greatly beneficial to optimizing compilation of nvcc for complex kernel with multi-loop hierarchy, where the innermost loop is related to variables that are known at runtime only. Like *Loop* C in MSV/SSV and Viterbi algorithm, *Q* is calculated as loop-count by Eq. 1 and can be pre-defined as a constant value in runtime compilation. As shown in Fig. 8, compiler is able to entirely unroll the *Loop* C with #pragma unroll*Q* to boost the performance. This is mainly because variables within innermost loop will be calculated and assigned to registers during compilation. However, for register-intensive kernel like P7Viterbi, loop unrolling leads to undesirable register spill. Thus, in contrast with MSV/SSV kernel, the loop-count *Q* is not passed as pre-defined constant to runtime compilation.

Furthermore, in order to obtain optimal performance, it is essential to dynamically switch between kernels best suited for the input problem. For smaller input query models, it is possible to store DP rows (alignment scores) within shared memory without sacrificing occupancy whereas for larger model sizes local memory is an optimal choice. Hence the application dynamically selects between the Shared and local memory implementation, the kernel with best configuration, to compile and run at runtime. This not only takes advantage of computing resources on device but also avoids performance degradation due to static configurations. Only the best suited kernel for the input problem is ever compiled and run, thus avoiding needless compilation of all kernels at compile-time with higher overhead. The cost of runtime compilation via NVRTC is listed in Table 2. As shown in the table, the maximum cost of compilation at runtime is around 800 ms which is negligible compared to total runtime of the application and may very well be completely hidden via multi-threading.

**Table 2:** Benchmark of elapsed time (ms) for each steps of runtime compilation

Steps of Runtime	MSV/SSV kernel		P7Viterbi kernel	
Compliation	Intel i5^a^	Intel Xeon^b^	Intel i5	Intel Xeon
Read.cu.cuh file into string	0.05/0.04	0.07/0.08	0.05	0.09
Create and compile nvrtcProgram	450/431	855/812	422	836
Get PTX string and kernel handle	0.35/0.77	5.38/5.63	0.27	7.64

^a^Intel i5-3570K quad-core 3.4 GHz CPU and 64-bit Ubuntu Linux

^b^Intel Xeon E5620 octa-core 2.4 GHz CPU and 64-bit Centos Linux

## Results and discussion

### Benchmark environment

In order to evaluate proposed MSV and Viterbi algorithms in CUDAMPF comprehensively, the benchmark analysis is composed of two parts: (1) the *intrinsic* comparison of different configurations in order to study the relationship between GPU kernel performance (GCUPS: GigaCell Update Per Second), cache hit ratio, kernel occupancy and the length of query models; (2) the *extrinsic* comparison of performance between CUDAMPF on GPU and hmmersearch from HMMER 3.1b2 on CPU.

In order to evaluate the scalability and performance, 24 different Hidden Markov Models of sizes ranging from 100 to 2405 were selected from 27.0 (released on May 2013) [35] with following accession numbers: PB000229, PB000603, PB002467, PB001249, PB000838, PB000131, PB001355, PB005588, PB014599, PB000340, PB000123, PB000768, PF05788.7, PB000358, PB001476, PB000744, PB000352, PB002016, PB000062, PB000265, PB003051, PF06317.6, PB000137, PB003055. As to protein sequence database, Swissprot database (released on July 2015) that contains 461414 protein sequences with totally 172 million residues were selected.

CUDAMPF running on single NVIDIA Tesla K40 with 15 SMXs (2880 CUDA cores) and 12 GB memory [36] was compared against HMMER MSVFilter, SSVFilter and ViterbiFilter running on a desktop workstation with an Intel i5-3570K quad-core 3.4 GHZ CPU, Intel i7-2600 octa-core 3.4 GHZ and on the single node of a compute cluster with an Intel Xeon E5620 octa-core 2.4 GHz server CPU, running 64-bit Linux operating system. Sequence scores of all three stages obtained from our implementation are completely identical to results of hmmersearch, and the number of sequences that pass through each filter is also matched.

### Intrinsic performance: NVRTC vs. static compilation

Tables 3 and 4 present various intrinsic parameters such as L1 Cache-hit ratio (for scoring DP matrix), Read-Only Cache-hit ratio (for model parameters) and register usage per thread and the overall performance (GCUPS) for HMM sizes ranging between 200 and 2405 and two different run-cases: static and NVRTC based compilation. The models were scored against the Swissprot database. The three intrinsic parameters, L1, Read-only cache hit ratio and register usage per thread, indicate the device memory utilization that impacts kernel performance directly. The maximum allowable number of registers per SMX (64 KB) was utilized by mounting 32 resident warps (1024 threads) where each thread was allocated 64 private registers. As shown in Table 3, for the MSV/SSV segment, NVRTC compiled kernels yield upto 2x and 3.2x faster in terms of GCUPS compared to statically compiled kernels respectively, due to the loop optimizations that utilize available registers as more as possible. The register usage per thread is always under 64 thus avoiding any register spill and the use of local memory.

**Table 3:** Performance comparison of static and runtime compilation for MSV/SSV kernel

	GCUPS		L1(%)		Read-only(%)		Register(64)^b^	
Model length	static	nvrtc	static	nvrtc	static	nvrtc	static	nvrtc
200	70/81	79/113	100/100	bypass/bypass^a^	100/100	100/100	42/33	32/30
600	118/146	165/235	100/100	bypass/bypass	98.3/98.3	98.1/98.1	42/33	44/30
1001	138/162	209/317	100/100	bypass/bypass	82.9/82.9	83/82.9	42/33	44/44
1400	146/165	239/367	99.9/100	bypass/bypass	74.3/74.3	74.2/74.2	42/33	45/44
2050	139/134	261/392	62.7/62.7	bypass/bypass	65.5/65.6	65.5/65.6	42/33	62/63
2405	139/138	277/440	58.3/58.7	bypass/bypass	63.6/63.5	63.7/63.9	42/33	63/62

^a^Sufficient private registers for each thread and no demand of local memory access

^b^The maximum number of available registers per thread

**Table 4:** Performance comparison of static and runtime compilation for P7Viterbi kernel

	GCUPS		L1		Read-only		Register	
Model length	static	nvrtc	static	nvrtc	static	nvrtc	static	nvrtc
200	9.7	8.7	99.9 %	43.4 %	99.1 %	99.1 %	62/64	spill^a^
600	12.3	4.9	55.9 %	3.9 %	85.7 %	86.3 %	62/64	spill
1001	10.4	4	52.4 %	2.9 %	74.5 %	75.2 %	62/64	spill
1400	9.6	3.3	50.3 %	1.8 %	68.6 %	69.8 %	62/64	spill
2050	9.3	2.9	49.4 %	0.5 %	62.7 %	62.6 %	62/64	spill
2405	10	2.9	49.5 %	0.4 %	61 %	61.4 %	62/64	spill

^a^Assigned private registers are exhausted. Registers spill to local memory

On the other hand, the complexity of P7Viterbi kernel requires higher number of registers per thread. Hence any loop optimizations performed by NVRTC causes severe register spill and limits the overall performance. The L1 cache-hit ratio degrades to 43.4 % even for short model length of 200 due to the use of local memory. Hence, for the P7Viterbi kernel the loop optimizations were not performed by keeping the inner-most loop count a runtime variable, thus achieving equivalent performance of statically compiled P7Viterbi kernel.

### Intrinsic performance: local memory vs. shared memory

**Fig. 9:**
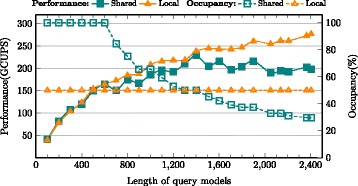
Performance comparison between MSV kernels of local memory and shared memory

**Fig. 10:**
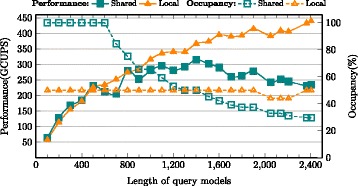
Performance comparison between SSV kernels of local memory and shared memory. The slight drop of occupancy curve for local memory is caused by NVRTC compilation

**Fig. 11:**
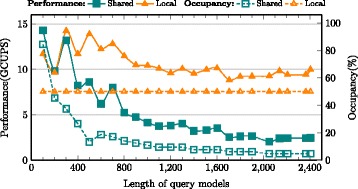
Performance comparison between P7Viterbi kernels of local memory and shared memory

The performance was evaluated for implementations of Shared memory vs. Local memory based storage of DP scoring matrix row. As mentioned in earlier sections, shared memory allocation limits number of active warps and the device occupancy as evidenced by Figs. 9, 10 and 11 for MSV, SSV and P7Viterbi respectively. The occupancy of MSV kernel declines from 100 % for a HMM size of 600 to 29.7 % eventually, which obviously degrades the performance. However, the local memory implementation exhibits a steady increase in performance while maintaining a constant device occupancy at 50 % irrespective of the size of the query model. Based on the benchmark (Swissprot database vs. 24 query models), only three smallest models of size 100, 200 and 300 showed slight advantage of shared memory over local memory, which are 40.86(vs. 39.28), 81.72(vs. 78.56) and 107.35(vs. 103.7) GCUPS respectively. SSV kernel has similar behaviours on performance between shared and local memory oriented implementations in which case larger models gain more speedup through using local memory (i.e, 1.87x faster than shared memory on model of 2405).

More pronounced performance gaps between two implementations were observed in P7Viterbi kernel. Since the usage of shared memory for P7Viterbi (due to *M/I/D* states) is about 3x that of MSV kernel, occupancy in this case degrades much more rapidly as does the performance. On the other hand, the local memory implementation yields upto 14.3 GCUPS for a model size of 300 and roughly maintains the performance at 10 GCUPS with increasing query model sizes. However, for small models with the length of 200 and below, shared memory kernel achieves slightly better performance.

### Extrinsic performance comparison: GPUs vs other processors

The original work [2] yields 12 GCUPS on MSVFilter and 1.6 GCUPS on ViterbiFilter by single core of an Intel processor. Acceleration via high-end FPGA designs in [15] yields upto 81 GCUPS for MSV and 3.6 GCUPS for P7Viterbi; GPUHMMER [6], an outdated acceleration with roughly 1.48 GCUPS, was modified by Lin [17] to accelerate MSVFilter in HMMER 3.x, which yields upto 32.8 GCUPS on a Quadro K4000 GPU. [20] implements cache-oblivious strategy to accelerate ViterbiFilter, that yields a roughly constant performance of 3 GCUPS on an Intel i7 processor and 1.7 GCUPS on AMD Opteron Bulldozer processor. [21] claims the first acceleration attempt for SSVFilter based on HMMER 3.1, which gains upto 372.1 GCUPS on a GTX570 GPU.

**Fig. 12:**
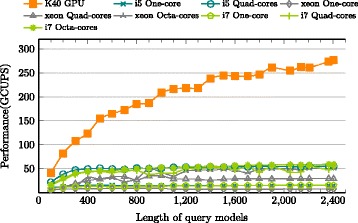
Evaluations of CUDAMPF with Intel Xeon, i5 and i7 on MSVFilter

**Fig. 13:**
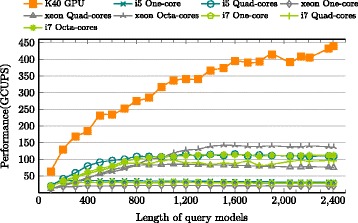
Evaluations of CUDAMPF with Intel Xeon, i5 and i7 on SSVFilter

**Fig. 14:**
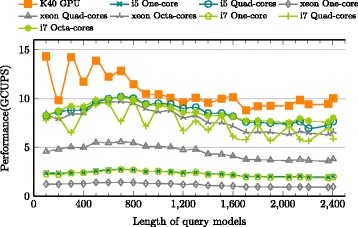
Evaluations of CUDAMPF with Intel Xeon, i5 and i7 on ViterbiFilter

The current implementation is compared to the latest version of hmmsearch in HMMER 3.1b2 on different processors. The comparison was performed by extracting and executing only the relevant methods of p7_pipeline for MSV, SSV and P7Viterbi segments. In order to monitor the execution time and CPU usage strictly, Intel Vtune Amplifier’s [37] hotspot profiler was used to measure the execution time of p7_MSVFilter, p7_SSVFilter and p7_ViterbiFilter separately for the entire sequence database. The baseline is measured in wall clock time, *T*_*last*_−*T*_*first*_, where *T*_*last*_ is time point after the last function call and *T*_*first*_ is time point before the first function call. Three high-end CPUs, Intel i5, i7 and Xeon, were evaluated with multiple cores as shown in Figs. 12, 13 and 14 for MSV, SSV and P7Viterbi stages respectively. On Intel Xeon, the average performances by utilizing single-, quad- and octa-cores are 7.7 (19.9), 27.2 (70.2) and 38.5 (103.7) GCUPS for MSVFilter (SSVFilter), meanwhile ViterbiFilter shows 1.1, 4.5 and 7.8 GCUPS. Similarly, Intel i7 gains 13.7 (29.1), 44.4 (80.4) and 50.5 (95.5) GCUPS for MSVFilter (SSVFilter) and 2.3, 7.4 and 8.6 GCUPS for ViterbiFilter, repectively. As to Intel i5, for MSVFilter (SSVFilter), we observed 14.5 (33.5) and 50.6 (98.8) GCPUS, and ViterbiFilter yields 2.3 and 8.6 GCUPS by employing single- and quad-cores. It can be seen that the performance of CUDAMPF clearly exceeds that of all implementations on CPUs and achieves a speedup of upto 37.5-fold (23.1-fold) over single core and 5.1-fold (3.2-fold) over multi-cores for MSVFilter (SSVFilter). Although P7Viterbi algorithm is more complex with strong dependencies, our method still runs 11.64x and 1.7x faster than single and multiple cores of CPUs, respectively.

## Conclusion

In this paper, we proposed a novel parallel framework CUDAMPF that embeds SIMD intrinsics within SIMT mechanism on CUDA-enabled GPUs, which greatly accelerate MSV/SSV and P7Viterbi stages of latest HMMER with 100 % accuracy, and the overall performance exceeds all other existing optimizations. In addition to the largely enhanced kernel throughput caused by synchronize-free execution, a finer-grained parallelism is achieved by this framework that could be also adopted to other similar problems in high-throughput sequence search. Based on the characteristics of the current algorithms, this work also presents an architecture-aware strategy to make optimal utilization of memory and cache system on the Kepler architecture for parallel efficiency and scalability. Moreover, CUDA Runtime Compilation (NVRTC) is incorporated to enable further optimization on kernel that wisely unrolls computational loops for performance boost, and it also support switchable kernels without compilation overhead in static. The strict performance evaluations illustrate that CUDAMPF gains significant speedup over CPU implementations: comparing with three different high-end CPU, our framework yields the maximum speedup of 37.5x (23.1x) and 5.1x (3.2x) over single and eight cores for MSV (SSV) kernel, and the P7Viterbi kernel gains 11.6x and 1.7x speedup, respectively.

*Future work:* The high-throughput sequence processing schema presented here will be integrated with heterogeneous computing enabled big-data processing framework running NoSQL database for indexed storage and retrieval of large omics data. The integrated framework will also be available for remote access via the world-wide-web.

## Availability and requirements

**Project name:** CUDAMPF**Project home page:**https://github.com/Super-Hippo/CUDAMPF**Operating system(s):** Linux**Programming language(s):** CUDA C/C++, PTX assembly**Other requirement(s):** CUDA 7.0 or later, GCC/G++ 4.4.7 or later, CUDA-enabled GPUs with Compute Capability of 3.5 or higher**License:** MIT License
